# Harmonized Medical Device Regulation: Need, Challenges, and Risks of not Harmonizing the Regulation in Asia

**DOI:** 10.4103/0975-1483.62221

**Published:** 2010

**Authors:** A Kaushik, KS Saini, B Anil, S Rambabu

**Affiliations:** *Department of Pharmaceutical Sciences, Jodhpur National University, Jodhpur, Rajasthan, India*; 1 *Medical Affairs, Johnson and Johnson Medical, Division of Johnson and Johnson Ltd, Gurgaon, Haryana, India*

**Keywords:** Harmonization, healthcare expenditure, democratic government, international organizations, international trade, reforms

## Abstract

Medical device sector is one of the most complex and challenging business segments of the healthcare industry with close collaboration between science and engineering. Despite the fact that Asia has 60% of the world population providing large market potential, Asian healthcare expenditure constitutes only 15% of the global healthcare expenditure. The accelerated ageing population and increasing prevalence of chronic disease are the key drivers that contribute toward the increase in the total healthcare expenditure on medical devices in the region. Several policies clearly showed the eagerness of the government to provide better healthcare infrastructure with better medical devices and facilities. The fundamental objective of the regulatory harmonization is to improve the efficiency of national economies and their ability to adopt to change and remain competitive. After the era of liberalization and globalization, the desires of developing economies is to ensure safety and performance of the product brought to their markets and for this harmonized regulation is an important tool for strengthening the same. If we talk about the industry need, then this approach will eliminate redundant requirements that do not contribute to safety and effectiveness. In addition, Asia is diverse in many respects and with it come the various challenges to harmonizing the regulation which includes diversity in culture, politics, economy, historical issues, etc. If, by any reason, the regulation of medical devices is not harmonized and consequently, the harmonized regulation is not adopted, then it leads to serious concerns like delayed or absent access to innovative technology, continued rise in the cost of medical therapies, etc. So this issue is written to attract all stakeholders to move toward the concept of harmonization, keeping in mind their need, challenges, and risks of not harmonizing the regulation as well.

## INTRODUCTION

The medical device sector is the fastest growing sector among its own category. Whenever a new medical device is developed, before it can be sold and supplied to the patient, it has to have a license from appropriate competent authorities worldwide. Since the beginning of 1980s, the regulatory world for medical devices has changed dramatically. From few countries, there are now 60-65 countries which have implemented regulation for medical devices or will soon implement the regulation for medical devices.[1]

First of all, I akin to initiate with what does the Medical Device Harmonization and Medical Device actually mean?

### Medical device harmonization

“To encourage convergence in regulatory practices related to ensuring the safety, effectiveness/performance and quality of medical devices, promoting technological innovation and facilitating international trade.”[2]

### Harmonization

“To bring into agreement or harmony.”

### Harmony *

“Agreement in feeling or opinion.”

(* According to the American Heritage College Dictionary, 3^rd^ edition; 2000)

Medical device sector is one of the most complex and challenging business segments of the healthcare industry with close collaboration between science and engineering. The term “medical devices” includes everything from highly sophisticated computerized medical equipment down to simple wooden tongue depressors. The intended primary mode of action of a medical device on the human body, in contrast with that of medicinal products, is not metabolic, immunological, or pharmacological.[2] But overall in broad sense we can describe medical devices as any instrument, apparatus, implement, machine, appliance, implant, *in vitro* reagent or calibrator, software, material or other similar or related article intended by the manufacturer to be used, alone or in combination, for human beings for one or more of the specific purposes of

- Diagnosis, prevention, monitoring, treatment, or alleviation of disease
- Diagnosis, monitoring, treatment, alleviation of or compensation for an injury
- Investigation, replacement, modification, or support of the anatomy or of a physiological process
- Supporting or sustaining life
- Control of conception
- Disinfection of medical devices
- Providing information for medical purposes by means of *in vitro* examination of specimens derived from the human body and which does not achieve its primary intended action in or on the human body by pharmacological, immunological, or metabolic means, but which may be assisted in its function by such means.[3]

## PRESENT SCENARIO OF THE MEDICAL DEVICE SECTOR IN ASIA

**Figure 1:**
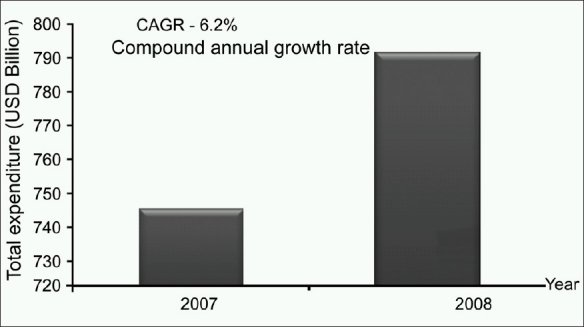
Total healthcare expenditure 2007-2008

Despite the fact that Asia has 60% of the world population providing large market potential, Asian healthcare expenditure constitutes only 15% of the global healthcare expenditure. In 2007, the total global healthcare expenditure was at US$ 4.981 trillion. With a growth rate of 6.2% as shown in Figure 1, the total healthcare expenditure of Asia was US$ 791.7 billion in 2008.[4]

**Figure 2:**
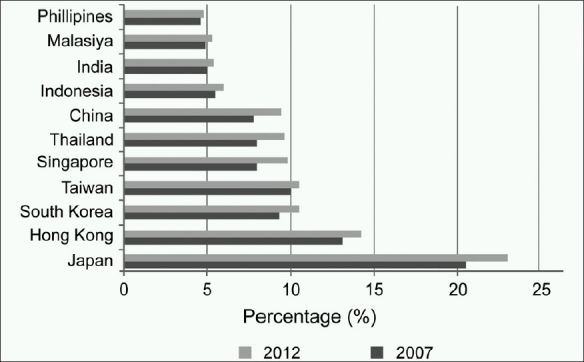
Percentage of population above 65 years, 2007-2012

While some parts of Asia are experiencing a high population growth, other countries such as Japan and China are facing the problem of accelerated ageing population. For instance, Japan is estimated to have 22.0% of its population above 65 years by 2012 as compared to 20.6% in 2007 [Figure 2]. With the current Asian lifestyle, the prevalence of chronic diseases such as diabetes, cancer, obesity and cardiovascular diseases has increased significantly. Other diseases that threaten the region include auto-immune diseases, infectious diseases, and neurological disorders. Accelerated ageing population and the increasing prevalence of chronic diseases are the key drivers that contribute toward the increase in total healthcare expenditure on medical devices in the region.[4]

Realizing the importance of disease prevention, the Asian governments invested billions of dollars annually to improve their healthcare infrastructure. For instance, the Ninth Malaysia Plan (2006-2010) is to work “toward achieving better health through consolidation of services” whereby emphasis has been placed on sustainability, upgrading, and maintenance of existing facilities and equipment, and improving the quality of healthcare. China announced its Healthy China 2020 plan in January this year, which aims to provide safe, effective, convenient, and low-cost public health and basic medical care to both rural and urban citizens by 2020.[4] Some key goals of the plan include making public medical institutions “nonprofit,” reducing the involvement of hospitals in the sale of drugs, increasing the role and responsibility of government, and establishing a basic medical care network for all Chinese citizens. High participation from private healthcare providers in developed countries in Asia promotes healthcare expenditure. For instance, Taiwan has fairly high participation of private healthcare, which contributes 65% of Taiwan’s total hospital beds. Private involvement has enabled efficient healthcare delivery to the people, which could be clearly seen in the case of Taiwan. This private participation was driven by Taiwanese comprehensive National Health Insurance scheme, which has eventually helped to increase the healthcare quality.[4] These policies clearly showed the eagerness of the government to provide better healthcare infrastructure with better medical devices and facilities.

## WHY WE REQUIRE HARMONIZATION?

A central function of any democratic government is to promote the economic and social well-being of its people. Governments seek to meet that objective in a wide variety of ways and in line with this, the regulation is an important tool that has helped governments make impressive gains in attaining desirable public policy goals. But where regulations impede innovation or create unnecessary barriers to trade, investment, and economic efficiency; duplication between regulatory authorities and different layers of government, and even among governments of different countries; the influence of vested interests seeking protection from competition; and regulations that are outdated or poorly designed to achieve their intended policy goals are all part of the problem. It is difficult to measure the precise cost of failure to harmonize the regulation, but it can be substantial. Delay heightens the cost of change in many cases. The hidden costs of inefficient regulation are increasing as the competition in global markets intensifies. In sectors characterized by rapid technological change or high international mobility, failure to harmonize the regulation can disadvantage entire economic sectors or lead to pressure for costly supports and protectionist policies. All governments have a continuing responsibility to review their own regulations and regulatory structures and processes to ensure that they promote efficiently and effectively the economic and social well-being of their people. A growing number of countries have embarked in recent years on ambitious programmes to reduce regulatory burdens and improve the quality and cost-effectiveness of regulations that remain. The difficulties and complexities have sometimes been greater than expected and there are many questions about the risks and costs of further reform, as well as continued strong opposition from vested interests.[5] Yet much has been learned about how to harmonize the regulation, and it is clear that the risks and difficulties of not harmonizing are often greater.

A fundamental objective of regulatory harmonization is to improve the efficiency of national economies and their ability to adapt to change and to remain competitive. Harmonization that sharpens competitive pressures provides powerful incentives to firms to become more efficient, innovative, and competitive. These improvements can boost the productivity of entire industries and often bring sharp and swift price reductions and improvements in the quality and range of products and services, to the benefit of consumers and user industries. For example, the European market alone, by promoting competition and replacing many separate national requirements by single Europe wide requirements, is estimated to have increased the European GDP by up to 1.5% between 1987 and 1993.[5] Harmonization that reduces business burdens and increases the transparency of regulatory regimes supports entrepreneurship, market entry, and economic growth that, in turn, should produce high-paying, high-quality jobs. Harmonization that reduces “red tape” and paperwork burdens for ordinary citizens — particularly in their role as taxpayers — frees up valuable time and individual initiative. In turn, more productive, innovative, and flexible economies are in a better position to meet other public interests and to help governments deal with issues such as social cohesion, environmental quality, and the rapid aging of populations.

## NEED FOR HARMONIZATION

After the era of liberalization and then globalization, international boundaries are no longer obstacles and the whole world is called as a Global Village. In addition if we concentrate little on changes in the world economy then we find that there is a slower growth in USA, EU, and Japan and robust growth in developing countries with sustained and rapid growth in China and India continuing their higher growth potential. There is a desire of developing economies to ensure safety and performance of products brought to their markets. Due to the competitive market, the pricing pressure is gradually increasing. Overall, the market trends prompt the need for a better operating model because traditional lines of business, technology, and competition are blurring; the definition of customer is changing; regulatory scrutiny is increasing; because of increasing regulatory pressure on governments and industry with scarcity of resources both human and financial; international market is big and getting bigger; and demographic trends are driving fundamental changes in the global economy.[5]

In our perspective, we can say that there is “internationalization” of the medical technology industry. There is global research and development, global clinical trials, global product introduction, multiple manufacturing location, etc. worldwide. Increasing worldwide recognition of medical device as a major future of healthcare industry sector - providing benefits for national independence for the medical device sector - is reflected in recent policy declaration by state as well as central government. For potential investors, however, global expansion of medical device continues to be viewed primarily through a financial and economic prism that focuses particularly on medical device’s competitiveness vis-à-vis other sectors of health industry such as pharmaceuticals and biologics. A major factor in this equation is the potential for economies of scale achieved by developing new medical devices in series. Currently, national variation in safety regulation presents an obstacle to internationally standardized regulation, which would faster these economies. The achievement of harmonization of medical device regulatory standard has overcome this obstacle, facilitating the emergence of a global market that offers a choice number of medical devices that are recognized by regulators as safe and technologically mature.

Adding to these, every regulatory body, while framing the regulation, has mainly two objectives that it wants to fulfill: First a timely and effective premarket review and second consistent and appropriate postmarket regulation. This can only be achieved when there is standardized regulation for medical devices which is internationally accepted.[5] This means that the whole process of premarket evaluation and/or postmarket surveillance takes as less time as possible because it saves time as well as investment to market the product that is prime requirement in the view of manufacturers.

There are various segments that are benefitted by this harmonization and we call it as harmonized regulation of medical devices. If we talk about industry’s need, then this approach will eliminate redundant requirements that do not contribute to safety. As far as regulators are concerned, they need the harmonized regulation because it reduces redundant reviews, creates an opportunity to share information on product safety, and results in a more efficient regulatory regime. The net result is improved trade in medical devices and safer products for consumers.

The harmonization of medical device regulation is needed to:[6]

1. Reduce the time to market the product by:
  - Eliminating undue or perhaps unjustified country-specific requirements
  - Providing transparent requirements which simplifies market access and expedites patients’ access to new and innovative technologies.
2. Reduce the cost to market the product by:
  - Establishing uniform international regulatory system and requirements
  - Reducing unwarranted, often contradictory regulatory requirements and redundant applications of similar requirements that can lead to different product definitions in various geographies, further complicating follow-up and comparison of data on safety issues which reduces resources and the complexity needed to meet local requirements and improves the utilization of already stretched and limited human and financial resources.
3. Improve government efficiency by:
  - Facilitating cooperation among regulators and the industry in conducting regulatory activities such as common audits, submission requirements, PMS procedures, acceptance and use of international standards, exchange of safety information which can be leveraged on experience gained over time.
4. Facilitate trade and expand market access by:
  - Creating common requirements for addressing the product life cycle (development, manufacture, placing on market, postmarket surveillance) which reduces the burden, complexity, and unpredictability for gaining market clearance.
5. Enhancement of public health protection by:
  - Establishing common and transparent premarket evaluation, postmarket surveillance, uniform quality system with similar audit criteria, and common clinical safety performance.

This provides increased product safety and efficacy thereby promoting public health and ensuring consumer confidence.

## CHALLENGES AND RISKS OF NOT HARMONIZING MEDICAL DEVICE REGULATION IN ASIA

The medical device industry growth in Asia is predicted to be extremely strong in the coming years. More technologies and products are being exported from Asia to the rest of the world. Asia is diverse in many respects and with it come the various challenges to harmonizing medical device regulations. A regulatory reform that enhances competition and reduces regulatory costs can boost efficiency, bring down prices, stimulate innovation, and help improve the ability of economies to adapt to change and remain competitive. Properly done, a regulatory reform can also help governments promote other important policy goals, such as environmental quality, health, and safety. Finally, country experience convinces us that the unavoidable disruptions which can accompany the reform can be addressed by complementary policies and actions.

Despite this, it is clear that many international organizations like GHTF, AHWP, APEC, ASEAN, etc. have made progress in developing standards and policies for the regulation of medical devices, particularly among their member nations. There is evidence of coordinated efforts such as the NCA Report Exchange Program, which has increased communication and dissemination of information between NCAs. However, it is questionable whether such progress has really resulted in any significant level of global harmonization. For example, 15 years after GHTF’s inception, its membership has not expanded beyond the original founding nations, and considering GHTF’s recognition of a “surging” increase in medical device manufacturing in nonmember nations, the scope and applicability of its harmonization policies is limited. Since the potential for medical devices manufactured outside member nations adhering to harmonized standard requirements is low, such harmonization efforts seem to be largely fruitless and it will be a long, slow road to global harmonization of medical device regulation.

One conclusion that emerges after reviewing the whole effort done by different organizations in different continents is that the most important ingredient for the successful harmonization of medical device regulation is the strength and consistency of support at the highest political level. Ministers have a direct role to play in assuring that strong political leadership will overcome vested interests in both public and private sectors which benefit from the *status quo* and resist beneficial change. But ministers and elected leaders cannot accomplish the necessary changes alone. Open dialog and communication is also an indispensable part of the process. It can strengthen the voices of those who support and will benefit from the harmonized guideline. Important allies in the harmonization process include businesses which will gain from low-cost, high-quality goods and service inputs; consumers; and employees and their representatives in fields in which job creation and wage growth are constrained by unnecessary regulatory restrictions.

If we talk about challenges for the harmonization of medical devices specifically, then we should consider that the following observations can be made:[6]

- Diversity in culture, language, politics, economy, race, religion;
- Differing regulatory capacity, expertise, infrastructure, finance;
- Varied collection of regulatory systems, differences in philosophy;
- Rising and varied expectation of medical technology;
- Government restrictions like control over the price; subsidy reduction involvement is a considerable challenge for any reform; due to account deficit the government may try to exclude a health insurance scheme in a particular area where a large investment of money is required;
- Local companies do not want to come under any regulatory body because they put him back to make unsafe devices;
- Historical issues — retention of sovereignty, trust, and confidence in decision/assessments from various regulators;
- Difficulties for stakeholders to reach consensus on harmonization efforts;
- Overall complexity of products and the market place;
- Sheer magnitude in the number of countries that are currently regulated, modifying regulations, developing new regulations;
- Many countries have long established regulatory systems which are difficult to change;
- Lack of trained manpower, lack of political commitment, and slow momentum in harmonizing fundamental issues such as definition, classification, nomenclature, and manufacturer.

According to the policy recommendation for reforms in regulation by OECD (Organization of Economic Co-operation and Development), there are different kinds of challenges for harmonization of medical devices that are[5]

- Adoption of a regulatory harmonization broad program at the political level that establishes clear objectives and framework for implementation;
- Reviewing regulation systematically to ensure that it continues to meet the intended objectives efficiently and effectively;
- Assurance that regulation and regulatory processes are transparent, nondiscriminatory, and efficiently applied;
- Reform of the regulations of medical devices at a global level in all sectors to stimulate competition and eliminate them except where clear evidence demonstrates that they have the best way to serve broad public interests;
- Identification of important linkages with other policy objectives and develop policies to achieve those objectives in ways that support harmonization.

Before discussing about risks of not harmonizing, I would like to introduce the words of *Lao Tzu* “If you don’t change the direction, then you may end up where you are heading,” which is self-explanatory that we must allow preexisting regulation of medical positive changes and of course this is also true with the devices which is country specific, i.e., not harmonized.

This means that we must harmonize the regulation for easy trafficking of devices throughout the world. If we concentrate particularly on India, then this becomes very important because:

- There are challenges of complying with disparate international regulatory systems and requirements regardless of the fact where the manufacturing hub is located;
- There are expending resources to deploy local infrastructure to access international markets;
- Regulatory authorities have to identify and comply with different sets of regulation for each targeted country;
- There are transparent and unified international regulatory systems to reduce time to market, i.e., seamless access.

So if we don’t harmonize the regulation, this will lead to:[7]

- Continued and escalating divergence between statutory regulatory systems leading to:
  1. Incomplete regulation, lack of implementing guidelines,
  2. Confounding regulation in following areas:
    - Premarket evaluation (product classification, submissions, review, clinical protocols/expectations, approval times)
    - Use of standards
    - Quality system requirements
    - Audits
    - Vigilance: Definition, reporting, decision making,
  3. Lack of or inability to leverage information sharing,
  4. Lack of common requirements/processes for handling new, innovative devices;
- Unpredictable environment due to the development of unclear regulation and control of the system;
- Continued rise in the cost of medical therapies;
- Uneven playing field leading to market access barriers;
- Delayed or absent access to innovative technology;
- Reduced, impaired access to important, life-saving devices.

## CONCLUSION

After considering all of the above-mentioned issues, we can say that when harmonized regulation of medical device comes into existence and consequently a uniform adoption of harmonized regulation takes place, then there is availability of quality product. People get assured about its effectiveness and safety. The small firms which produce fake and/or ineffective devices become obsolete from the market. These harmonized guidelines lead to the export-import of the product with less restriction worldwide and in other words we can say that there is a seamless access to the innovative medical device. But all of the above findings require a comprehensive research, meetings in order to exchange views and ideas of experts, gathering thoughts of legendry institutions, healthcare professionals and stakeholders, and definitely sharing the value with well-reputed healthcare firms. If all these things that we are considering above go in the right direction then we must configure a harmonized regulation for medical devices fulfilling the requirement of global standards. This all lead the Indian market with all of the above and the Vision-20 must be achieved.

## References

[1] Randeo Si (2006). Harmonization of Regulation in India: Benefits and challenges of Harmonization. 13^th^ AHWP-FICCI Workshop.

[2] WHO.int/en/.

[3] Ghtf.org.

[4] Asianhhm.com.

[5] Oecd.org.

[6] (2008). Albertson Carolyn. Harmonization of Regulation in India- Challenges of Harmonization. 13^th^ AHWP-FICCI Workshop.

[7] (2008). Albertson Carolyn. Harmonization of Regulation in India- Risks of not harmonizing the regulation. 13th AHWP- FICCI Workshop.

